# *In Vitro* Proliferation and Production of Cytokine and IgG by Human PBMCs Stimulated with Polysaccharide Extract from Plants Endemic to Gabon

**DOI:** 10.3390/molecules191118543

**Published:** 2014-11-13

**Authors:** Line Edwige Mengome, Aline Voxeur, Jean Paul Akue, Patrice Lerouge

**Affiliations:** 1 Institutde Pharmacopée et de MédecineTraditionnelles (IPHAMETRA), BP 1935 Libreville, Gabon; E-Mail: linemengome@yahoo.fr; 2 Centre International de Recherches Médicales de Franceville (CIRMF), BP 769 Franceville, Gabon; 3 Laboratoire Glyco-MEV, IRIB, Université de Rouen, 76821 Mont-Saint-Aignan, France; E-Mails: a.voxeur@gmail.com (A.V.); patrice.lerouge@univ-rouen.fr (P.L.)

**Keywords:** pectins, hemicelluloses, cytokines, IgG, human PBMCs, endemic plants, Gabon

## Abstract

Polysaccharides were extracted from seven plants endemic to Gabon to study their potential immunological activities. Peripheral blood mononuclear cell (PBMC) (5 × 10^5^ cells/mL) proliferation, cytokine and immunoglobulin G (IgG) assays were performed after stimulation with different concentrations of polysaccharide fractions compared with lipopolysaccharides (LPS) and concanavalin A (ConA) from healthy volunteers. The culture supernatants were used for cytokine and IgG detection by enzyme-linked immunosorbent assay (ELISA). The results show that pectin and hemicellulose extracts from *Uvaria klainei*, *Petersianthus macrocarpus*, *Trichoscypha addonii*, *Aphanocalyx microphyllus*, *Librevillea klaineana*, *Neochevalierodendron stephanii* and *Scorodophloeus zenkeri* induced production levels that were variable from one individual to another for IL-12 (3–40 pg/mL), IL-10 (6–443 pg/mL), IL-6 (7–370 pg/mL), GM-CSF (3–170 pg/mL) and IFN-γ (5–80 pg/mL). Only hemicelluloses from *Aphanocalyx microphyllus* produce a small amount of IgG (OD = 0.034), while the proliferation of cells stimulated with these polysaccharides increased up to 318% above the proliferation of unstimulated cells. However, this proliferation of PBMCs was abolished when the pectin of some of these plants was treated with endopolygalacturonase (*p* < 0.05), but the trend of cytokine synthesis remained the same, both before and after enzymatic treatment or saponification. This study suggests that these polysaccharides stimulate cells in a structure-dependent manner. The rhamnogalacturonan-I (RGI) fragment alone was not able to induce the proliferation of PBMC.

## 1. Introduction

Infectious diseases and immune-system diseases are still highly prevalent despite the modern treatments available (grafting, thymic injection, *etc.*). The immune system operates at two different levels: innate immunity and adaptive immunity. Innate immunity involves an unspecific factor or mechanism, such as the skin barrier, pH, enzymes, natural antibodies, certain cells or interleukin-like interferon-γ [1,2,3]. In contrast, adaptive immunity requires a reaction between a foreign body (antigen) and a receptor in the host. These receptors are carried by lymphocyte cells (TCR, BCR), which are divided into two families: B lymphocytes and T lymphocytes. However, the lymphocytes recognize the antigen only when an antigen presents cell-like macrophages or dendritic cells carrying a major histocompatibility complex (MHC) on its surface. This cell presents the antigens to lymphocytes to induce an immune reaction. It has been shown that the immune reaction can be polarized under stimulation. Therefore, under the influence of certain stimuli, the immune reaction can be oriented toward the Th1 subset, characterized by the prominent synthesis of cytokine-like interferon-γ, IL-2, *etc.*, or Th2, characterized by the prominent synthesis of IL-4, IL-5, IL-6 and IL-10 [4]. This suggests that the course of the immune response can be influenced by external factors. Plants have been used for many centuries [5,6,7], and the efficacy of plant decoctions has been shown in several reports. However, most of the studies on plants are based on their secondary metabolites, although a few studies have investigated the immunostimulant activities of plant polysaccharides [8,9,10]. However, because of their vast structural diversity, which suggests variability in their biological activities, further studies are needed to fully understand their biological properties. Furthermore, few studies have been carried out specifically on parietal polysaccharides from endemic plants in Gabon, the subject of this study.

## 2. Results and Discussion

### 2.1. Plant Samples

Four classes of plants endemic in Gabon were studied: Magnoliales, Ericales, Sapindales and Fabales (Table 1). Magnoliales is represented by *Uvaria klainei*, an Annonaceae only found in Gabon. The second class is Ericales, represented by *Petersianthus macrocarpus*, a member of the Lecythidaceae family that is widespread in tropical Africa (Guinea to Angola and the Democratic Republic of Congo). In Gabon, this species is found frequently in the north. It is used as an antiseptic, for cicatrizing and to treat lumbago and venereal disease. Sapindales is the third class represented by *Trichoscypha addonii*, an Anacardiaceae that has spread out from tropical Africa: its bark is usually used to treat dysentery and amenorrhea. Four Fabaceae-Caesalpinioideae in the Fabales class are represented by *Aphanocalyx microphyllus*, which is found throughout central Africa; *Librevillea klaineana* is only found in Gabon and is used to treat venereal disease; *Neochevalierodendron stephanii*, found in the equatorial forest, is used as an antibiotic; *Scorodophloeus zenkeri*, found throughout Africa, is used as a spice and treats high blood pressure and respiratory disease. The bark, stems or leaves of these plants are used (Table 1).

**Table 1:** List of plants endemic to Gabon investigated in this study. Please carefully check the table.

Angiosperm Phylogeny	Plants, Family and Names Used		Part and Names Used	Traditional Action	References
**Magnoliales**	*Uvaria klainei* Pierre ex Engl. & Diels; Annonaceae		Leaves (UkL)	-	-
			Stems (UkS)	-	-
**Ericales**	*Petersianthus macrocarpus* (P. Beauv.) Liben; Lecythidaceae		Barks (PmB)	Antiseptic, abortive, hypotensive	[11,12]
**Sapindales**	*Trichoscypha addonii* De wild; Anacardiaceae		Barks (TaB)	Dysentery, amenorrhea	[11]
**Fabales**	*Aphanocalyx microphyllus* (Harms) Weiring	**Fabaceae –Caesalpinioideae**	Barks (AmB)	-	-
	*Librevillea klaineana* (Pierre ex Harms) Hoyle		Barks (LkB)	Venereal disease	
	*Neochevalierodendron stephanii* A. Chev.) J. Léonard		Leaves (NsL)	Antibiotic	[11]
	*Scorodophloeus zenkeri* Harms		Barks (SzB)	Spice; treats high blood pressure, respiratory disease	

### 2.2. Isolation and Chemistry of Polysaccharides

**Figure 1:**
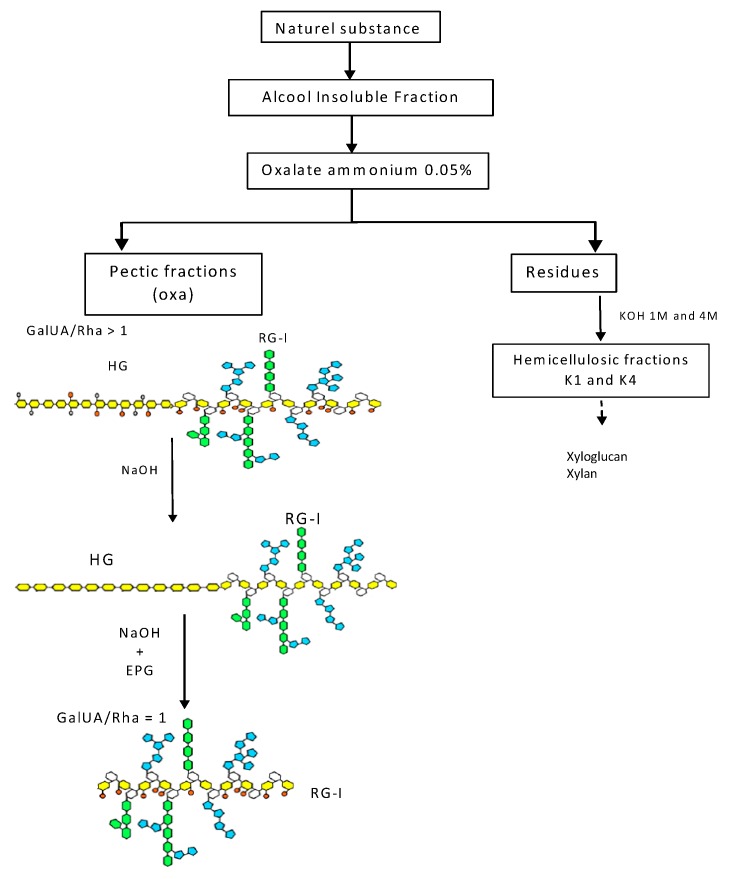
Scheme for the isolation of pectic and hemicellulosic fractions and the effects of endopolygalacturonase (EPG) and saponification by NaOH on the pectic material. Monosaccharides: galacturonic acid (GalUA), yellow; rhamnose (Rha), white; galactose (Gal), green; arabinose (Ara), blue; oxa, oxalate.

The nature and structures of polysaccharides isolated from the bark, leaves or stems of some of these endemic plants have been described elsewhere [13]. Briefly, the natural substances were heated in boiling 70% alcohol. Insoluble materials were then successively treated with ammonium oxalate and KOH 1 M and 4 M (Figure 1). The fractions solubilized with ammonium oxalate (called oxa) mainly contained pectic polysaccharides. Their sugar composition indicates that galacturonic acid (GalUA), rhamnose (Rha), galactose (Gal) and arabinose (Ara) are the main constitutive monosaccharides. As a consequence, these fractions contain homogalacturonan (HG), which is a polymer of repeated units of α(1-4)-d-GalUA that can be methyl-esterified and acetyl-esterified, and rhamnogalacturonan I (RG-I), which consists of the repeating disaccharide, α(1-4)-d-GalUA-α(1-2)-l-Rha, substituted with a wide variety of side chains attached to the rhamnosyl residues, ranging from monomers to large oligosaccharides, such as β(1-4)-d-galactan and α(1-5)-l-arabinan. The ratio between GalUA and Rha in pectic extracts varied between two and five, indicating that these fractions contained various proportions of HG and RG-I. To better characterize which part of these pectic polysaccharides was responsible for the activities observed, pectic fractions were either saponified with NaOH to remove methyl and acetyl ester groups linked to GalUA residues or saponified and then treated with an endopolygalacturonase (EPG) to remove HG chains (Figure 1) [13]. The sugar composition of the resulting enzyme-treated fractions indicated that the GalUA/Rha ratio is about one, as expected for a pure RG-I fraction [13].

Fractions solubilized by KOH 1 M (calledK1) and 4 M (K4) are mainly composed of xylose (Xyl) residues, indicating that they contained xylan and/or xyloglucans. In a previous study, the main polysaccharides of hemicellulosic fractions isolated from the leaves and stems of *Uvaria klainei* and from the bark of *Petersianthus macrocarpus* and *Aphanocalyx microphyllus* were identified as XXXG-type xyloglucans and β(1,4)-xylans substituted by 4-*O*-Me GlcUA residues [13].

### 2.3. Study Population

The cells from the population tested came from 12 individuals, both males and females (Table 2) with a mean age of 28 years with normal blood parameters, as commonly defined in normal individuals from this area, without any apparent clinical symptoms suggesting a chronic or acute disease.

**Table 2:** Physical and biological data of volunteers (*n* = 12); mean ± SD.

Averages	Men	Women
**Age (years)**	29.33 ± 3.1	27.67 ± 5.6
**Weights (Kg)**	74.17 ± 6.6	67.17 ± 10.2
**Heights (cm)**	171.5 ± 5.7	159.3 ± 3.4
**Temperature (°C)**	37.05 ± 0.3	37.1 ± 0.2
**Blood-pressure (mmHg)**	106.67/75	108.33/73.33
**Red corpuscle (mm^3^)**	4,323,333 ± 136,666.7	5,098,333 ± 301,666.7
**White corpuscle (mm^3^)**	3983.33 ± 783.3	6716.67 ± 1883.3
**Lymphocytes (mm^3^)**	1904.52 ± 314.1	2279.57 ± 699.4

### 2.4. Analysis of PBMC Proliferation under Polysaccharides Stimulation

**Figure 2:**
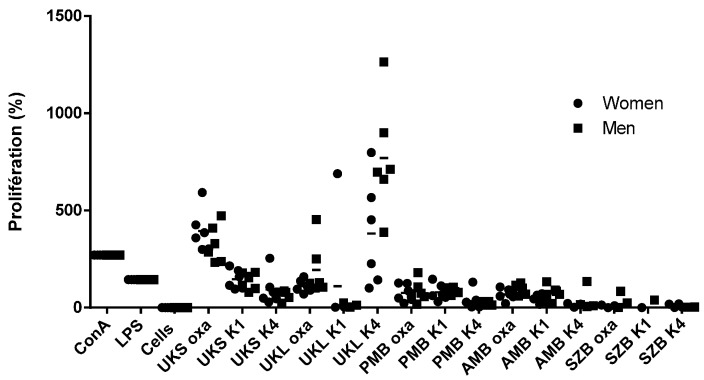
Proliferation of PMBCs from 12 volunteers stimulated by polysaccharide extracts: oxalate (oxa), 1 M KOH (K1) and 4 M (K4) compared with concanavalin A (ConA), lipopolysaccharide (LPS) and unstimulated cells; mean ± SD.

The proliferation tests carried out with the cells of the 12 volunteers (Table 2) showed different degrees of proliferation under polysaccharide stimulation and standard mitogens concanavalin A (ConA) and lipopolysaccharides (LPS). All 12 individuals had significant proliferative activity (*p* < 0.05) when stimulated with polysaccharides or standard mitogen (ConA and LPS) compared with the same unstimulated sample. The level of proliferation in the stimulated cells varied from 0% to 798% for females and from 0% to 1263% for males (Figure 2). The highest proliferations were observed with PBMCs stimulated with pectins from the stems and leaves of *Uvaria klainei* (UKSoxa, UKLoxa), and the bark of *Petersianthus macrocarpus* (PMBoxa) and *Aphanocalyx microphyllus* (AMBoxa) (Figure 2). However, the highest proliferation was also seen with PBMCs stimulated with hemicelluloses from the stems and leaves of *Uvaria klainei* (UKSk1, UKLk4) and hemicelluloses from the bark of *Petersianthus macrocarpus* (PMBk1) and *Aphanocalyx microphyllus* (AMBk1) (Figure 2). The variability of the response according to the stimuli suggests the diversity of the stimulating structure. The extracts from oxalate were more reactive, which is not surprising, considering that it has been shown that arabinogalactan type II (HG II) is an immunomodulator [9]. It is known that HG II comes from oxalate-digested extract. Other studies have shown the immunomodulating activity of polysaccharides [8,14,15,16]. However, this study is the first with plants endemic to Gabon, although polysaccharides from fragments rich in xylan acid extract from *Fleurya aestuans* (Linnaeus) in Gabon have shown increased proliferation of B-cells after stimulation with *Staphylococcus aureus* Cowan A [10].

**Figure 3:**
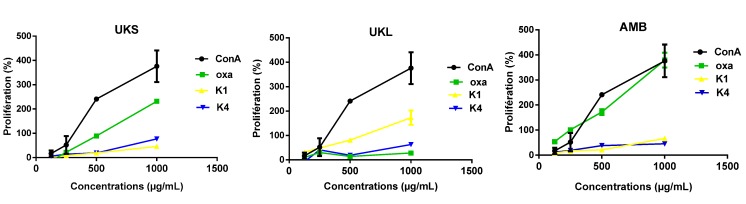
Representation of the dose-response effect of polysaccharide extracts: oxalate (Oxa), 1 M KOH (K1) and 4 M KOH (K4) compared with concanavalin A (ConA) (*n* = 6); mean ± SD.

In addition to the variability of the proliferative response, the level of proliferation is also dose-dependent, as shown in Figure 3 for the pectic fractions from *Uvaria klainei* (UKSoxa) and *Aphanocalyx microphyllus* (AMBoxa) and the hemicellulosic fractions of *Uvaria klainei* (UKSk1) leaves. It is also clear that within the same plant, the activity varies depending on the part used.

### 2.5. Analysis of Cytokine Synthesis 

**Figure 4:**
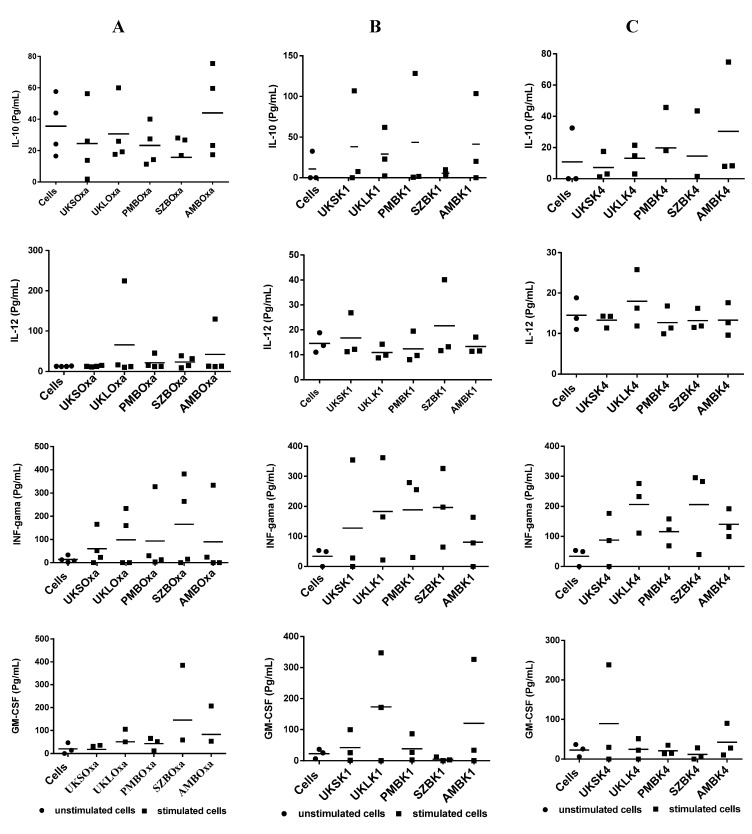
(**A**) Induction of cytokines by PMBCs from four volunteers after stimulation with pectin extracts compared with unstimulated cells; (**B**) profile of cytokine induction of PMBCs from three volunteers after stimulation by hemicellulose extracts (K1) compared to unstimulated cells. (**C**) Profile of cytokine induction of PMBCs from four volunteers after stimulation by hemicellulose extracts (K4) compared with unstimulated cells.

A set of PBMCs from the study’s subjects was used to measure the level of cytokine synthesis in the first step with pectin extracted from UKSoxa, UKS, UKLoxa, PMBoxa, SZBoxa and AMBoxa. The results (Figure 4A) show substantial variation at the individual level. This was observed with the synthesis of IFN-γ, which varied from 60.2 ± 73.5 pg/mL to 165.6 ± 188.7 *versus* 14.15 ± 13.77 pg/mL for unstimulated cells), IL-10 from 17.9 ± 12.93 pg/mL to 43.92 ± 28.1 pg/mL *versus* 35.58 ± 18.73 pg/mL for unstimulated cells and IL-12 from 12.65 ± 1.54 pg/mL to 65.7 ± 105.8 pg/mL *versus* 12.6 ± 0.56 pg/mL for unstimulated cells; then, GM-CSF from 22.5 ± 19.6 to 148.20 *versus* 20.67 ± 24 pg/mL for unstimulated cells. IL-6 was also elevated with the greatest amount of extract compared to unstimulated cells (7.97 to 323.63 pg/mL *versus* 37.54 pg/mL for unstimulated cells). However, the synthesis of IL-4 and IL-2 was not observed *in vitro*. Despite the differences at the individual level in the quantity of cytokine secreted by PBMCs stimulated by pectins, the difference with unstimulated cells as a group was not statistically significant (*p* varied between 0.38 and 1). The same experiment was conducted with PBMCs stimulated with hemicelluloses from K1 and hemicelluloses from K4. The results again show heterogeneity in the response, although the level of cytokine-like IFN-γ stimulated with SZBK1, UKLK4, PMBK4 and AMBK4 was sometimes very high (Figure 4B,C). These differences between stimulated and unstimulated cells did not reach statistical significance (197.4 pg/mL, 232.4 pg/mL, 121.9 pg/mL and 130 pg/mL, respectively, *versus* 49.5 pg/mL for unstimulated cells; *p* = 0.08 in all cases).

This study did not show any significant change in cytokine production before and after stimulation with the polysaccharides tested. The lack of IL-4 and IL-2 synthesis may stem from technical failure rather than complete absence of synthesis. However, the rationale of carrying out this experiment was based on the importance of cytokine in immunity [17,18,19] and suggested that the pooled sample from different individuals may not reveal the real trend in cytokine synthesis in a given study*.*

### 2.6. Effect of Chemical and Enzymatic Treatment on the Capacity of Immunostimulation by Polysaccharides

**Figure 5:**
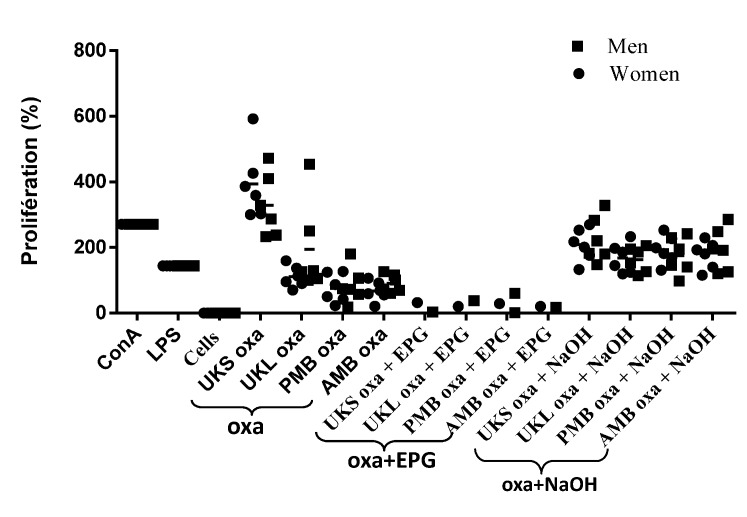
Proliferation of PMBCs from 12 volunteers stimulated by polysaccharide extracts: pectin (oxa), oxalate saponified (oxa + NaOH) or digested with EPG (oxa + EPG) compared with concanavalin A (ConA), lipopolysaccharide (LPS) and unstimulated cells; mean ± SD.

For this experiment, the PBMCs of the 12 subjects were tested after stimulation by pectins treated enzymatically with EPG and after saponification (NAOH) compared to unstimulated PBMCs from the same individuals and PBMCs stimulated with mitogens (LPS and ConA) (Figure 5). It appears that proliferation was abolished when pectin treated with EPG was used for stimulation of PBMCs, while proliferation was significantly high with PBMCs treated with saponified pectin (*p* < 0.05) compared to cells that were unstimulated or stimulated with EPG, regardless of the sex of the cells’ donors (*p* < 0.05).

**Figure 6:**
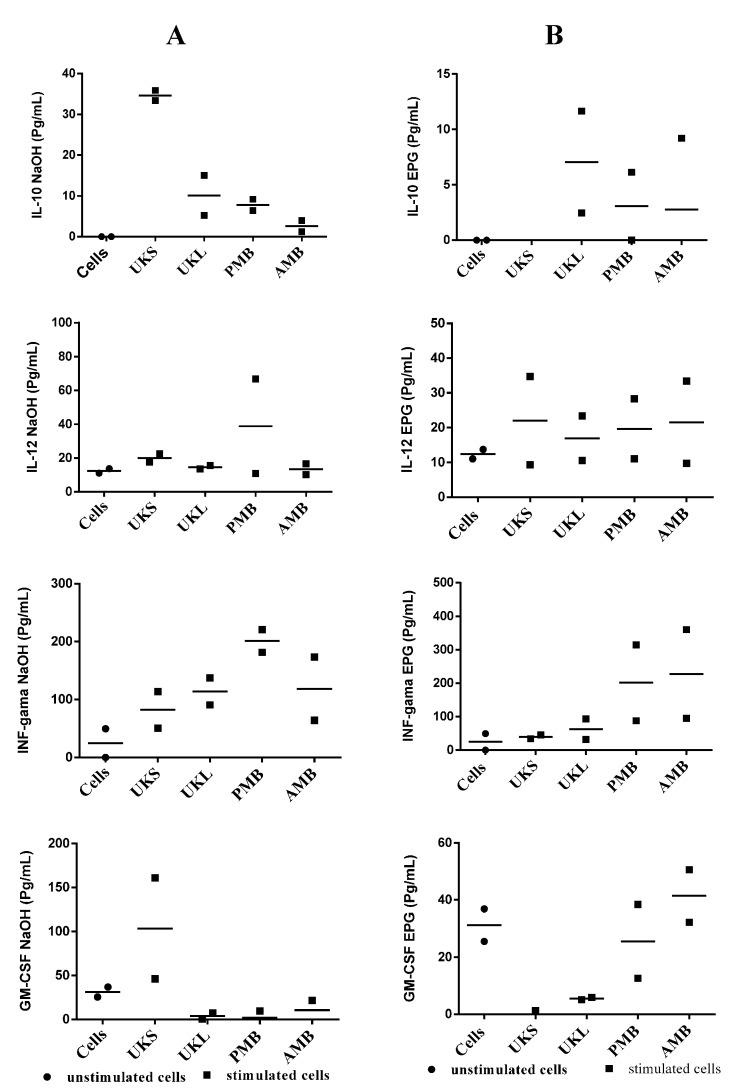
Representative dot scatter of the profile of cytokine induction by PMBCs of two volunteers after stimulation by saponified oxalate extracts (oxa + NaOH). (**A**) Oxalate extracts digested by EPG; (**B**) from plants (UKS, UKL, AMB) compared to unstimulated cells.

Similarly, cytokine secretion was examined after chemical and enzymatic treatment. As previously observed with cytokine synthesis with cells stimulated with untreated polysaccharides, the response remained heterogeneous with variation in the group of subjects, and the difference between cells stimulated by treated polysaccharides and unstimulated cells was statistically non-significant (*p* = 0.24 and 1) among groups treated with either NaOH or EPG (Figure 6). The substantial variation between individuals suggests that genetic characteristics are involved. It has been shown that TLR, which seems to be the receptor site for plant extract stimulation, can vary from one person to another. The heterogeneous nature of the allelic repertoire of human TLR has been shown [20]. An alternative explanation may be that the response to a specific extract is dependent on the immunological environment in each individual [21].

### 2.7. Analysis of IgG Secretion in Vitro

**Figure 7:**
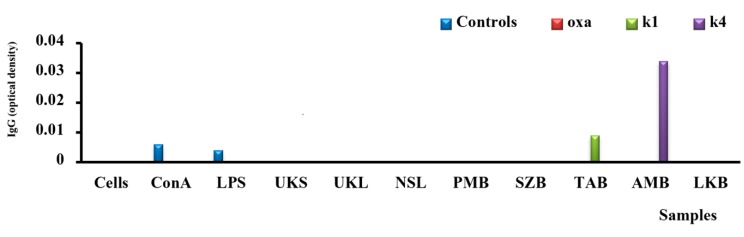
Illustration of the induction of IgG with polysaccharide extracts: oxalate (oxa), KOH 1 M (k1) and 4 M (k4) compared with concanavalin A (ConA), lipopolysaccharides (LPS) and unstimulated cells.

Immunoglobulin G (IgG) is the major immunoglobulin in the sera and accounts for approximately 70% of immunoglobulin. This isotype has several properties and valuable effector functions (fixation of antigen, complement, cells, neutralization, agglutination and precipitation). It can cross the placental barrier and the absence of IgG synthesis or its poor quality can lead to several diseases, such as allergic diseases and primary immune deficiency disease. In this study, only hemicelluloses from the bark of *Aphanocalyx microphyllus* (AMBK4) induced synthesis of IgG by PBMCs, as shown in Figure 7; in comparison with unstimulated cells or LPS- and ConA-stimulated cells. However, the subclass was not determined. Similar results were obtained with *Echinacea angustifolia* extract, which was not only capable of increasing the production of specific IgG in chickens immunized with human albumin, but was also capable of restoring the synthesis of IgG in an immunodeficient chicken. Other studies have confirmed these observations, showing that administration of ginseng extract induces an increased production of specific IgG to antigen administered after treatment with ginseng extract. Such results open new perspectives in the search for new treatments for atopic allergies, autoimmune disease and primary immunodeficiency (PID).

In general, studies on the relationship between pectin structure and biological activities are not new. However, the diversity and the complex nature of the structure of these molecules preclude generalization of their activity to all plant-derived polysaccharides; thus, this assertion substantiates our study on these specific Gabonese plants. Many studies have shed light on the importance of pectin, which appears as very bioactive polysaccharides [22]. In our study, although the mechanism by which the pectin tested is acting on cells proliferation was not studied, the first aim was to demonstrate that these molecules have an effect on PBMC; further study should analyze the mechanism underlying PBMC proliferation under polysaccharide stimulation. Previous work has shown the importance of pectin and their modified forms [22], and some mechanisms of action have been described, which involved binding and inhibition of galectin-3 (GAL-3) via the RG-I domain of polysaccharide. GAL-3 is a protein that is overexpressed in the cancer state and is present on many cells’ surface and in the extracellular environment. Galectin-3 has been shown to modulate T-cell growth [23]. It also regulates monocyte and macrophages function [24]. Because removal of the HG region by enzymatic treatment in our experience suppress PBMC proliferation, it is likely that maintaining the HG and RG-I domain together may be important to maintain cell proliferation activity in this study. Evidence of the activity of pectin on immune modulation is provide by the experiment on β glucan [25,26]. Other experiments have shown that polysaccharides from *Biophytum petersianum* with β-d-(1→4)-galactan containing a side chain in their RG-I region play an important role in immunomodulation against Peyer’s patch cells [27]. It has been suggested that polysaccharides bind to a specific receptor on the cell [28], and it has been shown that soluble β-glucan polysaccharides bind to the lectin site of neutrophils or natural killer cells’ CR3 [29]. That orally-delivered glucan may inhibit infection [30], that oligosaccharides can alter regulatory T-cell function against vaccine responsiveness [31] and the diversity observed in the polysaccharides structure offer numerous perspectives in the health sciences.

## 3. Experimental Section

### 3.1. Natural Samples

The therapeutic properties of the plants selected were reviewed [11,32,33]. The plants were collected in the Estuaire and Ngounie regions of Gabon. Each voucher specimen was deposited in the National Herbarium of Gabon (HNG). Different parts were used for extraction: leaves, bark and stems. These parts were dried and crushed at the Institut de Pharmacopée et de Médecine Traditionnelle (IPHAMETRA) in Gabon. The polysaccharides were isolated and characterized at the University of Rouen in France. The immunomodulator activities of the samples were recorded at the Centre International de Recherches Médicales de Franceville (CIRMF), Gabon.

### 3.2. Human Samples

Blood samples were obtained from human volunteers from whom informed consent was obtained (mean age, 28 years; healthy male and female individuals). Ethical clearance was obtained from the National Ethical Committee of Gabon, Reference PROT No. 0006/2013/SG/CNE. Blood samples were drawn from the arm in uncoagulated form and used for isolation of PBMCs and plasma.

### 3.3. Polysaccharides from Cell Wall Extraction and Characterization

Dried plant organs were ground and agitated in boiling water for 1 h to obtain a water-insoluble sample. These samples were then incubated in methanol for 24 h. The insoluble material was collected and then agitated in boiling ethanol 70% for 1 h. The alcohol-insoluble residues, mainly composed of cell wall polymers, were submitted to sequential chemical extraction, as previously described [13]. Briefly, the residues were boiled in 0.05% ammonium oxalate for 1 h. The soluble extracts, called oxa, were separated by centrifugation, dialyzed against water and freeze-dried. The pellets were then incubated in potassium hydroxyl 1 M (Fraction K1) and 4 M (Fraction K4) containing 20 mM NaBH_4_. The monosaccharide composition of these fractions was determined by gas phase chromatography analysis of the trimethylsilyl methylglycoside derivatives according to [34]. The pectic fraction was demethyl-esterified (oxa + NaOH) and digested with endopolygalacturonase (EPG).

### 3.4. Analysis of PBMC Proliferation

PBMCs from healthy human volunteers were isolated using the Ficoll gradient after centrifugation at 1500 rpm/min for 30 min, washed three times with phosphate buffered saline (PBS), pH 7, and then distributed in 96-well plates as a suspension of 5 × 10^5^ cells/mL in an RPMI medium containing 10% fetal calf serum and penicillin/streptomycin. The plates were then incubated in an incubator at 37 °C in a 5% CO_2_ atmosphere for 24 h before adding different polysaccharide extracts at a 0- to 1000-µg/mL concentration and mitogen (ConA, LPS at 0–1,000 µg/mL). The plates were again left to incubate for 4 days. At this stage, MTT was added to each well and left to incubate for 2 h in the same conditions. Then, 50 µL of isopropanol were added and mixed with the cells. Plates were then passed through a spectrophotometer at 570 nm, and the optical density (OD) of each well was recorded. Then, the percentage of proliferation was calculated as % = (OD sample − OD control) × 100, where the OD sample is the one obtained 5 days after stimulation by polysaccharides or mitogens, and the OD control was obtained with unstimulated cells after 5 days in culture.

### 3.5. Analysis of Cytokines Synthesis 

The following cytokines were measured *in vitro* according to the manufacturer’s protocols (Quantikine R&D Systems, Abingdon, U.K.): IL-2, IL-4, IL-6, IL-10, IL-12, GM-CSF, IFN-γ. Briefly, PBMCs from healthy volunteers were cultured at 1 × 10^6^/mL in similar conditions as for the proliferation test. They were left in culture for 24 h before the addition of 100 µL of different polysaccharides and mitogens to 24-well plates. This mixture was left for 3 days in culture before harvesting the supernatant, which was used for cytokine measurement. We incubated 100 µL of each supernatant in a precoated well with a monoclonal against each specific cytokine. The rest of the protocol followed the manufacturer’s instructions. The quantity of cytokine was determined using a curve plotted with the known quantity of cytokine against the OD measured at 450 nm. The sensitivity limit was 7 pg/mL, 10 pg/mL, 0.7 pg/mL, 3.9 pg/mL, 5 pg/mL, 3 pg/mL and 5 pg/mL for the detection test of IL-2, IL-4, IL-6, IL-10, IL-12, GM-CSF and IFN-γ, respectively.

### 3.6. Analysis of the Secretion of IgG

This was performed according to the methods commonly used for the measurement of the IgG level in the culture supernatant [35,36] with some modifications. Briefly, a 96-well microtiter plate was coated with 100 µL of a monoclonal antihuman IgG at 10 µg/mL in carbonate buffer, pH 9.6, overnight. The following day’s plate was washed three times with 100 µL of TBS/ Tween 0.05%, then incubated for 2 h with 5% bovine serum albumin (BSA) in TBS/Tween 0.05%, washed again and incubated with 100 µL of supernatant obtained from the PBMC culture that had been stimulated in the same manner as for the cytokine assay. After this, the incubation, plate was washed again three times with TBS/Tween 0.05%, followed by a 1-h incubation with a polyclonal antihuman IgG labeled with alkaline phosphatase diluted to 1/2000 in TBS/Tween 0.05% with 1% BSA. The reaction was demonstrated by the incubation of each well with 100 µL of phosphatase substrate for 30 min at room temperature. The OD was measured at 405 nm in a spectrophotometer. *In vitro* secreted IgG was determined as OD stimulated cells minus OD unstimulated cells.

### 3.7. Statistical Analysis

The mean level plus or minus the standard deviation and median were determined for each experiment carried out in triplicate or for each data group. The nonparametric Mann–Whitney *U*-test was used to compare the difference in proliferation or cytokine level among the groups. These analyses were performed with Minitab 16 software (Minitab Inc., State College, PA, USA). A *p*-value less than or equal to 0.05 was considered significant.

## 4. Conclusions

This study confirms the immunostimulatory properties of polysaccharides. The limits of these properties appear to be related to their molecular structure and the nature of the host. In this specific case, the removal of the HG backbone abolished the PBMC proliferation. In addition, the polysaccharides studied do not all act in the same way: some affect the production of effectors (antibodies, cell proliferation), but the production of cytokines by different individuals under stimulation was heterogeneous. It is therefore necessary to study the detailed structure of these molecules in relation to their immunostimulatory activity.
